# A Health Survey of African American Men Seen at an Academic Medical Center in the Southern United States

**Published:** 2021-09-13

**Authors:** Steven S Coughlin, Deepak Nag Ayyala, Justin Xavier Moore, Ban A Majeed, Marlo M Vernon, Hayat Dergaga, John S Luque

**Affiliations:** 1 Department of Population Health Sciences, Medical College of Georgia, Augusta University, Augusta, GA.; 2 Institute of Public and Preventive Health, Augusta University, Augusta, GA.; 3 Department of Medicine, Augusta University, Augusta, GA.; 4 Georgia Cancer Center, Augusta University, Augusta, GA.; 5 Department of Psychology, Augusta University, Augusta, GA.; 6 Institute of Public Health, Florida Agricultural and Mechanical University, Tallahassee, FL.

**Keywords:** African Americans, Cardiovascular disease, Congestive heart failure, Diabetes, Hypertension, men, Prostate cancer, Stroke

## Abstract

**Background:**

African Americans have poorer cardiovascular health and higher chronic disease mortality than non-Hispanic whites. The high burden of chronic diseases among African Americans is a primary cause of disparities in life expectancy between African Americans and whites.

**Methods:**

We conducted a cross-sectional study via a postal survey among a sample of 65 male, African American patients aged ≥ 40 years. The overall objective was to examine the frequency of high blood pressure, high cholesterol, diabetes, myocardial infarction, congestive heart failure, stroke, asthma, emphysema, and cancer among patients treated at Augusta University Health.

**Results:**

A high percentage of study participants (81.5 %) reported a history of high blood pressure; 50.8% had high cholesterol; 44.3% were overweight, 44.3% were obese, and 13.9% were current cigarette smokers. About 36.9% of the men had a reported history of diabetes; 10.8% of the men had a history of heart attack, 13.9% had a history of congestive heart failure, 9.2% had a history of stroke, and 15.4% had a history of prostate cancer. Men who reported a personal history of prostate cancer were significantly more likely to have a history of heart attack and stroke and to be overweight (p < 0.05 in each instance).

**Discussion:**

Additional studies are needed of cardiovascular risk factors and adverse cardiovascular events among African American men, and interventional research aimed at controlling hypertension. Of particular concern is prostate cancer, and whether patients with hypertension, hypercholesterolemia, and diabetes are receiving appropriate therapy to reduce their cardiovascular risk and prevent morbidity and mortality from adverse cardiovascular events.

## Introduction

Life expectancy in the U.S. in 2015 was 75.7 years for African Americans compared to 79.2 years for whites [1]. This disparity in life expectancy, which is particularly pronounced for African American men, is mainly driven by marked racial disparities in leading causes of death such as heart disease and diabetes [1]. African Americans also experience pronounced disparities in cancer mortality [2–6]. African Americans have poorer cardiovascular health and higher Cardiovascular Disease (CVD) mortality when compared to non-Hispanic whites. The high burden of CVD among African Americans is a primary cause of disparities in life expectancy between African Americans and whites [7]. The higher prevalence of CVD risk factors (e.g., hypertension, diabetes mellitus, and obesity) underlies the relatively earlier age of onset of CVDs among African Americans. Hypertension is highly prevalent among African Americans and contributes to disparities in stroke, heart failure, and peripheral artery disease [7]. The prevalence of diagnosed and undiagnosed hypertension among African American men (42.4%) greater than 20 years of age in the U.S. is among the highest in the world [7].

African American men develop diabetes mellitus 1.52 times more often than white men [7]. The combined prevalence of diagnosed and undiagnosed type 2 diabetes mellitus is 21.8% in African Americans and 11.3% in non-Hispanic whites [8]. African Americans are twice as likely to die from diabetes as non-Hispanic whites [9]. In a study of racial disparities in diabetes mortality in the 50 most populous U.S. cities, African Americans had statistically, significantly higher mortality rates compared to whites in 39 of the 41 cities included in the analyses [9].

Obesity rates are higher among African Americans than whites. Among adults 20 years of age and older, African American women had the highest rates of obesity at 58%, followed by African American men (38%), white men (34%), and white women (33%) [10]. Factors including greater prevalence of nutrient poor diets contribute to obesity and obesity-related chronic diseases like CVD and hypertension [11,12].

Prostate cancer is the most commonly diagnosed cancer in the U.S. [13]. African American men have the highest prostate cancer incidence rates in the world and the highest prostate cancer mortality rate of any racial/ethnic group in the U.S. [14]. African American men are more likely to have locally advanced or metastatic prostate cancer at diagnosis, present at an earlier age, and have suboptimal outcomes to standard treatments [15]. African American men are 2.5 times more likely to die from prostate cancer than white men [13].

Due to the ongoing disparities in cardiovascular comorbidities among African American men, we conducted a study among a sample of African American male patient’s ages 40 years and older to examine the frequency of chronic diseases and chronic disease risk factors. The overall objective was to examine the frequency of high blood pressure, high cholesterol, diabetes, myocardial infarction, congestive heart failure, stroke, asthma, emphysema, and cancer among patients see at Augusta University Health, a large academic medical center in the Southern U.S. In addition, there was interest in cancer comorbidity including self-reported prostate cancer.

## Methods

Data are from the African American Men’s Health Survey, a cross-sectional study of male, African American patients seen at Augusta University Health. Potential respondents were randomly sampled using electronic medical records. Non-institutionalized men were eligible to take part in the study if they were at least 40 years of age and resided in Augusta-Richmond County or Columbia County, Georgia, or in Aiken County, South Carolina.

Data were collected using postal survey questionnaires. The mailings were sent to 1,000 potential research participants. A sequential mailing protocol was followed using a modified Dill-man method. An advance letter was mailed to the men by the study principal investigator (SSC). The letter provided information about the study (purpose, potential benefits, and risks). Three weeks later, a survey consent letter was mailed to those who had not opted out along with a copy of the survey questionnaire and a pre-addressed, stamped return envelope. Those who had not opted out or returned a completed questionnaire were sent a reminder postcard three weeks later.

We obtained outcome measures through a self-reported postal survey which included questions about chronic disease risk factors and diseases (congestive heart failure, high blood pressure, diabetes, myocardial infarction, stroke, asthma, emphysema, and cancer). Respondents were asked, “Please mark an X beside each of the health conditions that you discussed with a healthcare provider.” Also, “Are you currently taking medications for any of the conditions below? (diabetes, high blood pressure, high cholesterol).” Body Mass Index (BMI) was estimated using self-reported information about height and weight. A BMI of 25–29.99 kg/m^2^ was defined as overweight; a body mass index greater than or equal to 30 kg/m^2^ was defined as obese. Survey responses were checked for completeness and then coded and entered into an electronic database.

Descriptive analyses were used to examine the frequency of chronic disease outcomes. We compared the distribution of comorbidities between men with a history of prostate cancer to those without a history of prostate cancer using Fisher’s exact test. We considered α = 0.05 as the level of significance level.

## Results

A total of 65 men completed the study questionnaires, corresponding to a response rate of 6.5%. The mean age of the men was 64.4 years (Table 1). Of the 65 surveyed participants, the majority of participants had two persons living within a household (n = 35, 59.3%), were retired (n = 28, 46.7%), were married or with partner (n = 41, 65.1%), had a high school educational level (n = 16, 25.4%), and reported good general health (n = 33, 53.4%).

A high percentage of the study participants (81.5 %) reported a history of high blood pressure; 50.8% had high cholesterol; 44.3% were overweight, 44.3% were obese, and 13.9% (9 of 65) were current cigarette smokers (Table 2). About 36.9% of the men had a reported history of diabetes. About 10.8% of the men had a reported history of heart attack, 13.9% had a history of congestive heart failure, 9.2% had a history of stroke, and 15.4% had a history of prostate cancer (Table 2).

Table 3 displays the results stratified according to whether or not the respondent reported a history of prostate cancer. Men who reported a personal history of prostate cancer were significantly more likely to have a history of heart attack and stroke and to be overweight (Table 3).

## Discussion

The results of this survey indicate that African American men seen at a large academic medical center in the Southern U.S. have a high prevalence of cardio-metabolic risk factors and disease including hypertension, hypercholesterolemia, diabetes, overweight and obesity. Hypertension is a major cause of cardiovascular morbidity and mortality and all-cause mortality. About 30% of all deaths among African Americans are attributable to hypertension [16]. Compared with whites, hypertension in African Americans is more prevalent, occurs earlier in life, is more severe, and is more often associated with cardiovascular complications [26,17]. African Americans have a greater burden of hypertension-related diseases (stroke, coronary heart disease, heart failure, and renal dysfunction) than the general population, which may be due to racial differences in peripheral vascular resistance, greater salt sensitivity, and circulating renin levels [16, 18]. Pathophysiologic mechanisms of hypertension among African Americans may involve the intra-renal renin-angiotensin system, variations in angiotensinogen, and increased aldosterone sensitivity [17]. There are also possible gene-environment pathways connecting psychosocial stress from direct or indirect experiences of racism with hypertension outcomes [19]. Cardiovascular disease is the leading cause of death among African Americans [16]. The rate of stroke mortality is higher in the southeastern region of the U.S. (the “stroke belt”) than in other regions [16].

In the current study, a high percentage of participants who reported having a history of hypertension also reported that they were taking antihypertensive medication. However, no information was available about whether the hypertension was controlled or not. African Americans are more likely to have uncontrolled hypertension than whites, which is likely to be partly due to level of adherence to blood pressure lowering medications [20]. Other contributors to uncontrolled hypertension include lack of knowledge about the condition, lack of support, having hypertension that is resistant to treatment, and comorbidities such as diabetes and renal disease [20].

Although avoidance of cigarette smoking, reduced obesity, and use of health care services can improve health, such factors are influenced by social determinants of health such as income, employment, and education [1, 2]. In our study among African-American men, we observed that 15% were living with less than $20,000 of annual income. Prior studies report that poverty rates are two times higher among African-Americans (25.4%) compared to non-Hispanic whites (10.4%) [1]. Unemployment rates are more than two times higher among African Americans compared to non-Hispanic whites [1]. There are also substantial disparities in educational attainment. Fewer African Americans graduate from high school (72.5%) than non-Hispanic whites (87.2%) [2].

About 25.4% of the men in current study reported a personal history of prostate cancer. A statewide study determined that African Americans in east central Georgia-an area near Augusta, Georgia-exhibited the greatest cancer disparities for prostate cancer in men compared to whites [21]. African American men are more likely to develop aggressive prostate cancer, yet less likely to be screened despite guidelines recommending shared decision-making about prostate cancer screening and PSA testing [11,15]. It is unclear whether screening through PSA testing reduces mortality [22]. However, no firm conclusions about the benefits-to harm ratio of PSA screening can be drawn in African American men due to their limited representation in two landmark clinical trials of the effectiveness of prostate cancer screening in reducing mortality from the disease [11]. A more recent study that used Surveillance, Epidemiology, and End Results (SEER) data to investigate survival disparities between African American and white men provided a compelling case for continued PSA testing for African American men [23]. The controversy surrounding prostate cancer screening, coupled with the high rates of incidence and mortality among African American men, make it that much more important for African American men to engage in an informed decision-making process around prostate cancer screening [24]. Previous studies have suggested that men who are more knowledgeable about prostate cancer are more likely to have been screened [25]. While informed decision-making is the current recommendation for prostate cancer screening, recent studies indicate that many African American men may not be making informed decisions about prostate cancer screening [11]. This is partly due to patients having limited knowledge of prostate cancer screening which could be improved by having these discussions with their healthcare provider [26]. In the current study, men who reported a personal history of prostate cancer were significantly more likely to have a history of heart attack and stroke and to be overweight.

With respect to limitations, we lacked information about the clinical characteristics and timing of prostate cancer. Misclassification bias is a possibility due to the use of self-reported information. However, the validity of self-reported hypertension has been reported to be high among African Americans [27]. A further issue is that the results of this study may not be generalizable to other populations of African American men. However, the sample was diverse in terms of socioeconomic factors and other demographic characteristics.

In conclusion, African American patients seen at medical centers such as Augusta University Health may have a high burden of cardio-metabolic risk factors and disease. Efforts may be needed to assist the effectiveness of clinical care teams, decrease care barriers, and improve patient adherence [28]. The relatively high number of patients with a personal history of prostate cancer suggests that further research is needed to examine the quality of prostate cancer survivorship care in this high-risk patient population.

## Figures and Tables

**Table 1:** Characteristics of study participants, African American Men’s Health Survey (n=65).

Characteristic	Frequency (%)
**Mean age** (SD)^1^ – years (*N* = *58*)	64.44 (9.32)
**Annual Income** (*N* = *60*)	
<$20,000	9 (15.0)
$20,000–$34,999	4 (6.7)
$35,000–$49,999	8 (13.3)
$50,000–$64,999	9 (15.0)
$65,000–$79,999	11 (18.3)
$80,000 +	8 (13.3)
Missing^2^	11 (18.3)
**Number of people in household** (*N* = *59*)	
1	13 (22.0)
2	35 (59.3)
3 +	11 (18.7)
**Employment status** (*N*=*60*)	
Retired	28 (46.7)
Employed	10 (16.7)
On disability	16 (26.7)
Temporarily unemployed	6 (10.0)
**Marital status** (*N*=*63*)	
Married/Partner	41 (65.1)
Single	12 (19.1)
Widowed	3 (4.8)
Separated/Divorced	7 (11.1)
**Education** (*N*=*63*)	
Less than HS	8 (12.7)
HS or equivalent	16 (25.4)
Some college	15 (23.8)
Associate degree	7 (11.1)
Bachelor degree	9 (14.3)
Graduate degree	8 (12.7)
**Perceived general health** (*N*=*63*)	
Excellent	1 (1.6)
Very good	8 (12.7)
Good	33 (52.4)
Fair	16 (25.4)
Poor	5 (7.9)

SD=Standard Deviation

**Table 2:** Self-reported History of Chronic Disease and Risk Factors among African American men (n=65).

Chronic disease/Risk factor	Frequency (%)
Heart attack	7 (10.8)
Congestive heart failure	9 (13.9)
Stroke	6 (9.2)
Diabetes	24 (36.9)
High blood pressure	53 (81.5)
High cholesterol	33 (50.8)
Prostate Cancer	10 (15.4)
**Cigarette smoking status**	
Current smoker	9 (13.9)
Former smoker	26 (40.0)
Never smoked	30 (46.2)
**Body mass index**	
Underweight	1 (1.6)
Normal weight	6 (9.8)
Overweight	27 (44.3)
Obese	27 (44.3)

**Table 3:** Self-reported History of Chronic Disease/Risk Factors among African American Men with and without a Personal History of Prostate Cancer (n=65).

Chronic disease/Risk factor	No Prostate Cancer Frequency (%) (N = 55)	Prostate Cancer Frequency (%) (N = 10)	p-value^1^
Heart attack	3 (5.5)	4 (40.0)	0.0085
Congestive heart failure	8 (14.5)	1 (10.0)	-
Stroke	3 (5.5)	3 (30.0)	0.0421
Diabetes	18 (32.7)	6 (60.0)	0.1537
High blood pressure	43 (78.2)	10 (100.0)	0.2489
High cholesterol	25 (45.5)	8 (80.0)	0.0824
**Cigarette smoking status**			
Current smoker	9 (16.4)	0 (0.0)	0.3263
Former smoker	20 (36.3)	6 (60.0)	
Never smoked	26 (47.3)	4 (40.0)	
**Body mass index**			
Normal weight	6 (10.9)	0 (0.0)	0.0224
Overweight	19 (34.6)	8 (80.0)	
Obese	26 (47.3)	1 (10.0)	

1 p value determined using Fisher’s exact test.
